# An analysis of characteristics and associated factors for re-contacts after EMS non-conveyance: a retrospective cohort study in the Netherlands

**DOI:** 10.1186/s13049-025-01365-8

**Published:** 2025-04-17

**Authors:** Renate F. Speijers, Remco H. A. Ebben, Ties Eikendal, Lobke Ruijs, Franciscus G. M. H. M. Cuppen, Rien de Vos

**Affiliations:** 1 Emergency Medical Service, Public Health and Safety Region Gelderland-Zuid, Nijmegen, The Netherlands; 2 Emergency Medical Service, Public Health and Safety Region Gelderland-Midden, Arnhem, The Netherlands; 3 Department of Emergency Medicine, Radboud University Medical Center, Nijmegen, The Netherlands; 4 Department of Emergency Medicine, Canisius Wilhelmina Hospital, Nijmegen, The Netherlands; 5 Department of Education and Training, Amsterdam University Medical Center, Amsterdam, The Netherlands

**Keywords:** Non-conveyance, Emergency medical services, Ambulances, Re-contacts

## Abstract

**Background:**

Non-conveyed patients are a significant population within ambulance care. To gain insight in patient safety for this population, ambulance re-contacts within 72 h are monitored. However, little is known about the background of these non-conveyance cases with a re-contact. This study aims to investigate the incidence of re-contacts, analyse characteristics, and identify factors associated with re-contacts following non-conveyance.

**Methods:**

This was a retrospective cohort study of all non-conveyance cases and all associated re-contacts in two EMS regions in the Netherlands, Gelderland-Zuid and Gelderland-Midden. Data was collected from 1 January 2022 till 31 December 2022. Characteristics of non-conveyance cases with and without a re-contact within 72 h were compared and differences were analysed univariately. Logistic regression analyses were used to quantify bivariate and multivariable associations between characteristics of non-conveyance cases and EMS re-contact within 72 h. Associations are expressed in odds ratios with 95% confidence interval.

**Results:**

In the analysis of 19.563 cases, the overall incidence for an EMS re-contact within 72 h was 5.0% (N = 984/19.563), with 3.4% (669/19.563) within 24 h, 1.0% (195/19.563) within 24–48 h and 0.6% (120/19.563) within 48–72 h. In a subset of 13.010 complete cases, significant multivariable associations were observed between re-contacts and age > 65 (OR 2.15, CI 1.82–2.53), male gender (OR 1.39, CI 1.18–1.63), and medical complaints related to specialism 'Pulmonology' (OR 2.45, CI 1.67–3.64), 'Neurology' (OR 1.81, CI 1.28–2.59) and 'Traumatology/surgery’ (OR 0.51, CI 0.34–0.76). Other significant associations were noted with night-time cases (OR 1.49, CI 1.21–1.82) and cases in which consultation or handover to a general practitioner occurred (OR 1.25, CI 1.06–1.47).

**Conclusions:**

A low overall incidence of EMS re-contacts indicates that non-conveyance within the EMS system is relatively safe. The likelihood of re-contact is higher for age above 65, male gender, and medical complaints within the specialisms of 'Pulmonology' and 'Neurology'. Non-conveyance cases that involve consultation or handover to a GP and cases occurring at night are also more likely to have a re-contact. The findings inform non-conveyance decision-making, and could serve as a starting point to adapt EMS curricula, and develop guidelines and protocols. This may fuel the enhancement of non-conveyance decision-making, thereby improving the quality of healthcare within the EMS system.

## Background

The past decades Emergency Medical Services (EMS) have changed from conveyance providers into providers of advanced pre-hospital care [1]. Along with this change the demand for emergency care has increased due to a variety of reasons e.g., population growth, aging of the population, changing triage systems, changes in the accessibility in general practitioner (GP) care and an increasing demand for patient self-management with a decrease in social support [1–3]. Within these developments non-conveyance has become a substantial and increasing part of ambulance care [4]. ‘Non-conveyance’ is defined as an appropriate ambulance deployment where the patient after on-scene assessment and/or treatment does not require transportation to a healthcare facility with medical personnel and equipment [5]. Non-conveyed patients are assessed, treated and discharged at the scene and can also be referred to other primary care facilities, such as the GP [6]. International non-conveyance rates vary between 3.7 and 93.7% in the general population [5]. For EMSs in the Netherlands the rate is approximately 25% [7]. Ambulance professionals perceive the non-conveyance decision as complex due to many influencing factors and patient safety may be compromised [8, 9]. Non-conveyed patients have been shown to have an increased risk for subsequent events compared with patients who had been conveyed and discharged from emergency departments (ED’s), such as EMS re-contacts (6.1% vs. 1.8%,), ED visits (4.6% vs. 1.4%,), hospital admission (3.3% vs. 0.8%) and even death (0.2% vs. 0.1%) [10]. In contrast, several studies indicate that non-conveyance is relatively safe and an appropriate triaged decision that leads to appropriate use of limited ambulance sources and reduces ED overcrowding [11–13].

Non-conveyance assessment and decision making requires guidance through guidelines and protocols, which are currently being developed [14–16]. To develop these guiding instruments insight into the non-conveyed patient population is needed. Previous studies showed that non-conveyed patients are generally younger and more often have reasons for care related to mental, behavioural and neurodevelopmental disorders in comparison with conveyed patients [13, 17–19]. Older age was found to be a risk factor for subsequent and adverse events [20, 21]. Although these studies give insight in the non-conveyed population, more insight is needed in that part of the non-conveyed population which has an ambulance care re-contact. Especially as re-contacts are frequently used as quality indicator for non-conveyance [22]. Additional information on characteristics associated with subsequent contacts and/or adverse events, as well as the relation between first and subsequent contact, may help further detailing of guidelines and protocols. A recent study in New Zealand identified age, sex and event type as associated factors for an EMS re-contact within 48 h [23]. In the Netherlands EMS re-contacts following non-conveyance have not been studied before.

## Methods

### Aim

The aim of this study was to investigate the incidence of EMS re-contacts following non-conveyance, to analyse the characteristics of non-conveyance cases with a re-contact with respect to patient-, care process- and run characteristics, and to identify which characteristics are independently associated with re-contacts following non-conveyance.

### Design

This was a retrospective cohort study of non-conveyance cases with and without a re-contact within 72 h. The 72 h limit is based on the Dutch national quality indicators for non-conveyance, measuring ambulance re-contacts within 24 h or 72 h after non-conveyance.

The General Data Protection Regulation (AVG in Dutch) and the Medical Treatment Agreement Act (WGBO in Dutch) were applied, and the study was approved by the board of both EMSs. Approval by the regional ethical committee (METC) was not applicable, because this study was not subject to the Medical Research Involving Human Subjects Act. However, the study did comply with the applicable ethical standards for scientific research following the Research Code Amsterdam UMC [24]. This study is reported following the STROBE-guidelines [25].

### Setting

This study was conducted in two EMS regions in the Netherlands: EMS Gelderland-Zuid and EMS Gelderland-Midden. EMS regions Gelderland-Zuid and Gelderland-Midden are two of the 25 EMSs in the Netherlands. EMS Gelderland-Zuid and Gelderland-Midden provide ambulance care for 541.000 people and 668.000 people respectively. Ambulance care can be requested via the national emergency number 112 or by other health professionals (medical specialist or the general practitioner). Ambulances are dispatched through regional emergency medical dispatch centres. At time of this study ambulances in both EMSs were dispatched by the same regional dispatch centre using the Dutch Triage Standard. Ambulance care can be dispatched with urgency level A1 (arrival < 15 min), A2 (arrival < 30 min), and B (not urgent, mostly planned ambulance care). Based upon type of request and location an ambulance or a rapid responder is dispatched. Ambulances are fully equipped and staffed with an ambulance professional (a nurse of a bachelor of health) and a driver. Rapid responders are solo vehicles (car or motorcycle) with one ambulance professional. Ambulance professionals are either registered nurses with additional training in emergency care, intensive care or cardiac care who followed a specific national training course in ambulance care for seven months, or bachelors of health, who have completed a four-year dedicated bachelor’s degree in ambulance care. Ambulance drivers are trained but do not have a nursing or medical degree and have an assisting role in medical care. Ambulance professionals are trained to work according to the national EMS protocol [26]. The national EMS protocol covers a range of emergency medical conditions and provides guidance on assessment and treatment of patients. Although consulting a physician is possible, the EMS protocol allows ambulance professionals to make decisions without direct consultation of a physician. After non-conveyance patients can contact their GP or the Ems system. There is no option to contact a private doctor or private EMS.

### Population

Included were all patients that were assessed and/or treated by an ambulance professional and then discharged at scene, including the subgroups of non-conveyed patients with and without an EMS re-contact within 72 h after the initial contact. Excluded were the non-conveyance ambulance deployments involving patients who were found dead or died during treatment at the initial EMS contact.

### Data collection

**Fig. 1:**
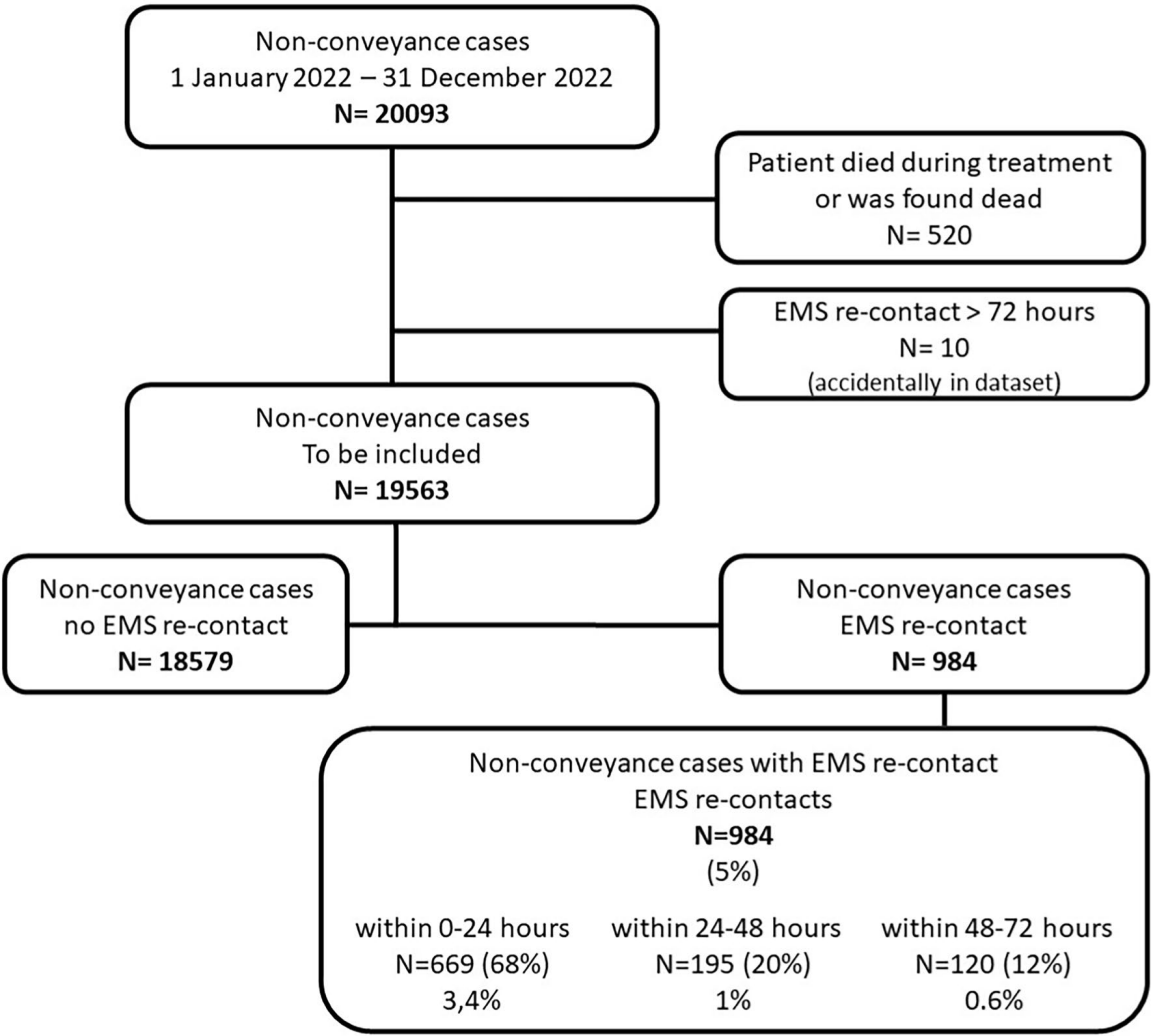
Inclusion of non-conveyance cases and re-contacts

Data were collected from 1 January 2022 till 31 December 2022from the electronic databases of EMSs Gelderland-Zuid and Gelderland-Midden. Each ambulance case handled by the dispatch centre is stored in those EMS databases and has an unique identification number. For this study all non-conveyance cases and all associated EMS re-contacts within 72 h were extracted and checked against the inclusion criteria (Fig. 1). All included non-conveyance ambulance cases were categorised in two subgroups: non-conveyance cases with a re-contact and non-conveyance cases without a re-contact. In accordance with classifications used in other studies [20, 23] and the Dutch quality signal [27], the incidence of the re-contacts was categorised into three time intervals: 0–24 h, 24–48 h and 48–72 h. We collected and presented variables similar to previous research [13, 17, 18, 23]. For each ambulance case, characteristics of the patient, the care process and the run of the initial (first) contact were collected. Patient characteristics involved age and gender. The characteristics of the care process involved medical specialism, consultation and/or handover to a GP and if the initial non-conveyance decision was patient (refusal of care) or professional initiated. Characteristics of the run involved urgency level, time, date and applicant. Age was collected as a continuous variable and additionally categorised into three groups according categorisation also used in several other studies: 0–17, 18–64 and > 65. Time of day was categorised, and based on the office hours of general practices and outpatients’ clinics in the Netherlands during the study period: day, 08:00–17:00 h; evening, 17:01–23:59 h; and night, 00:00–07:59 h. Based on the calendar date, we extracted the variables day of week and month of year, and we categorised months of the year into the four meteorological seasons. Medical specialism is registered in the EMS database according to codes in the basic set of ambulance care [28] and collected as such. For each re-contact (second contact) characteristics of the run (urgency level, time, date and applicant) and the care process (medical specialism) were collected. Based on time and date of the initial and second contact, we extracted the time between the initial contact and the re-contact. We defined the outcome of the re-contact as non-conveyance, admission to the emergency department (ED) or a Cardiac Care Unit (CCU), outpatient clinic, hospitalisation and death.

### Statistical analysis

The incidence of re-contacts was analysed using descriptive statistics and presented as numbers and percentages for three time intervals: within 24 h, 24–48 h and 48–72 h.

To identify characteristics that are associated with a re-contact, we described and compared the two subgroups (with and without re-contact) during the initial (first) ambulance contact. Analysis of these patient-, care process- and run characteristics was done with ambulance cases with complete data on the variables age, gender and medical specialism, using descriptive statistics and presented as numbers, percentages, mean and Sd, median and interquartile range (IQR). Differences between the two subgroups were analysed using the Mann Whitney U test, Fisher exact test and Pearson Chi-square test. All variables that had a significant difference with a *P* < 0.05 between both groups were eligible for logistic regression analysis. First in unadjusted logistic regression, bivariate associations between the dependent variable and each independent variable were determined and expressed as unadjusted ORs with 95% CI. Second, variables that were bivariate statistically associated with re-contact were entered into an adjusted multivariable logistic model to determine the independent contribution to a re-contact. Adjusted associations were expressed as adjusted ORs with their 95% CI.

Characteristics of the re-contact (second contact) were analysed for re-contacts with complete data on the variables age, gender and medical specialism using descriptive statistics and presented as numbers and percentages. The re-contact outcome was analysed for all re-contacts. Data analysis was performed using R (V4.3.0). A *P*-value < 0.05 was considered statistically significant for all statistical analysis performed.

## Results

### Incidence

A total of 20,093 non-conveyance cases were identified, 530 cases were excluded for not meeting the inclusion criteria. The remaining non-conveyance cases (n = 19,563) were included to calculate incidence rates. The overall incidence for a re-contact within 72 h was 5.0% (N = 984/19563). Incidence rates for a re-contact in the three time intervals were 3.4% (669/19563) within 24 h, 1.0% (195/19563) within 24–48 h and 0.6% (120/19563) within 48–72 h.

### Characteristics of non-conveyance cases with and without an EMS re-contact, Initial contact

Of the 19,563 non-conveyance cases used for calculating the incidence rates, 6553 cases with incomplete data on the variables age (N = 38), gender (N = 65) and medical specialism (N = 6450) were omitted for further analysis, leaving 13,010 non-conveyance cases. Complete cases did not differ from the group with missing data on age and gender. The proportion of missing data was equivalent in both groups with and without a re-contact. Table 1 shows the patient-, care process- and run characteristics of the initial non-conveyance cases with (N = 661) and without a re-contact (N = 12,349).

**Table 1:** Characteristics of non-conveyance cases with and without an EMS re-contact

Variable	Non-Conveyance with an EMS re-contact N = 661	Non-Conveyance without an EMS re-contact N = 12,349	*P*-value
Patient characteristics			
Age (mdn, IQR)	67 (46.80)	51 (27.71)	*P* < 0.001
Age category			
0–17	42 (6%)	1560 (13%)	
18–64	267 (40%)	6689 (54%)	
> 65	352 (53%)	4100 (33%)	
Sex			*P* < 0.001
Male	373 (56%)	6047 (49%)	
Female	288 (44%)	6302 (51%)	
Care process characteristics			
Medical specialism			*P* < 0.001
Cardiology	55 (8%)	992 (8%)	
Surgery/traumatology	59 (9%)	2727 (22%)	
Pulmonology	59 (9%)	437 (4%)	
Neurology	88 (13%)	1051 (9%)	
Internal medicine	303 (46%)	5199 (42%)	
Pediatrics (including neonatology)	22 (3.5%)	539 (4.5%)	
Psychiatry (including panic disorders)	71 (11%)	1362 (11%)	
Gynecology/obstetrics	4 (0.5%)	42 (0.5%)	
Consultation/handover to a GP			*P* < 0.001
Yes	274 (41%)	4141 (34%)	
No	387 (59%)	8208 (66%)	
Patient refusal of care			*P* = 0.187
Yes	9 (1%)	103 (1%)	
No	652 (99%)	12,249 (99%)	
Run Characteristics			
Urgency level			*P* = 0.003
A1 (arrival < 15 min)	465 (70%)	7985 (65%)	
A2 (arrival < 30 min)	196 (30%)	4364 (35%)	
B (planned ambulance care)	0	0	
Applicant			*P* = 0.003
Citizen/112	334 (50.5%)	6063 (49%)	
General practitioner	24 (3.5%)	410 (3.3%)	
Out-of-hours GP service	104 (15.5%)	1551 (12.5%)	
Other healthcare professional^1^	21 (3%)	348 (3%)	
Non-healthcare professional^2^	10 (1.5%)	93 (0.7%)	
Unknown	168 (25%)	3884 (31.5%)	
Time of day			*P* < 0.001
Day 08:00–17:00;	267 (40%)	5566 (45%)	
Evening, 17:01–23:59;	217 (33%)	4462 (36%)	
Night, 00:00–07:59	177 (27%)	2321 (19%)	
Day of week			*P* = 0.384
Monday	89 (13%)	1806 (15%)	
Tuesday	86 (13%)	1686 (14%)	
Wednesday	81 (12%)	1760 (14%)	
Thursday	101 (15%)	1661 (13%)	
Friday	100 (15%)	1604 (13%)	
Saturday	103 (16%)	1970 (16%)	
Sunday	101 (15%)	1862 (15%)	
Season			*P* = 0.975
Winter	158 (24%)	2975 (24%)	
Spring	173 (26%)	3182 (26%)	
Summer	162 (25%)	3110 (25%)	
Autumn	168 (25%)	3082 (25%)	

^1^Healthcare institution, Obstetrician, Psychiatrist

^2^Fire department, Police

With respect to patient characteristics, the group with a re-contact had a significant higher median age in years (67 vs. 51, *p* < 0.001) and a significant higher proportion of males (56% vs. 49%, *p* < 0.001), compared to the group without a re-contact.

As for the care process characteristics there were statistically significant differences in medical specialism and consultation or handover to a GP between the group with and without a re-contact. In cases with a re-contact the proportions were higher for medical complaints related to the medical specialism ‘Pulmonology’ (9% vs. 4%), ‘Neurology’ (13% vs. 9%) and ‘Internal Medicine’ (46% vs. 42%), whereas the proportion of medical complaints related to ‘Trauma’ was lower (9% vs. 22%) (*p* < 0.001), compared to cases without a re-contact. In the initial non-conveyance cases which were followed by a re-contact, consultation or hand over to a GP occurred more frequently, compared to the initial non-conveyance cases without a re-contact (41% vs. 34%, *p* < 0.001). There was no significant difference in patient refusal of care or conveyance between both groups.

Regarding run characteristics, there were significant differences in urgency level, applicant and time of day between both groups. Non-conveyance cases with a re-contact had a significant higher urgency level (70% vs. 65%, *p* = 0.003) compared to non-conveyance cases without a re-contact and the non-conveyance cases during the night did have more re-contacts at a later time (27% vs. 19%, *p* < 0.001). In non-conveyance cases with a re-contact, the applicant was more often an out-of-hours GP service (15.5% vs. 12.5%), compared to non-conveyance cases without a re-contact. There were no significant differences in day of the week and season.

### Characteristics associated with EMS re-contact

Multivariable logistic regression analyses showed that there were significant independent associations between patient-, care process- and run characteristics of the initial non-conveyance cases and re-contact within 72 h (Table 2). As for patient characteristics, the likelihood of a re-contact for patients aged > 65 years was considerably higher compared to patients aged 18–64 years (OR 2.15 CI 1.82–2.53). Also, male gender increased the likelihood for a re-contact (OR 1.39 CI 1.18–1.63).

**Table 2:** Characteristics associated with EMS re-contact; results from logistic regression

Variable	Unadjusted Odds Ratio * (95% CI)	Adjusted Odds Ratio ** (95% CI)
Patient characteristics		
Age category		
0–17	**0.67 (0.48–0.93)**	**0.61 (0.37–0.95)**
18–64 (ref)	**–**	–
> 65	**2.15 (1.82–2.53)**	**2.11 (1.78–2.51)**
Sex		
Male	**1.35 (1.15–1.59)**	**1.38 (1.18–1.63)**
Female (ref)	**–**	–
Care process characteristics		
Medical specialism		
Cardiology (ref)	–	–
Surgery/traumatology	**0.39 (0.27–0.57)**	**0.51 (0.34–0.76)**
Pulmonology	**2.44 (166–3.58)**	**2.45 (1.66–3.63)**
Neurology	**1.51 (1.07–2.15)**	**1.81 (1.28–2.59)**
Internal medicine	1.05 (0.79–1.43)	1.17 (0.88–1.60)
Pediatrics (including neonatology)	0.74 (0.44–1.22)	1.90 (0.96–3.78)
Psychiatry (including panic disorders)	0.94 (0.66–1.35)	1.42 (0.98–2.08)
Gynecology/obstetrics	1.72 (0.50–4.44)	3.07 (0.88–8.17)
Consultation/handover to a General Practitioner		
Yes	**1.41 (1.19–1.65)**	**1.25 (1.06–1.47)**
No (ref)	–	–
Run Characteristics		
Urgency level		
A1 (arrival < 15 min) (ref)	–	–
A2 (arrival < 30 min)	0.77 (0.65–0.92)	1.07 (0.88–1.29)
Applicant		
Citizen/112 (ref)	–	–
General practitioner	1.06 (0.68–1.59)	0.85 (0.53–1.29)
GP out-of-hours service	1.22 (0.97–1.50)	0.99 (0.77–1.25)
Other Healthcare professional^1^	1.95 (0.94–3.60)	1.81 (0.86–3.40)
Non-healthcare professional^2^	1.09 (0.67–1.68)	1.45 (0.88–2.26)
Unknown	0.79 (0.65–0.95)	0.78 (0.64–0.95)
Time of day		
Day 08:00–17:00; (ref)	–	–
Evening, 17:01–23:59;	1.01 (0.84–1.22)	1.02 (0.85–1.24)
Night, 00:00–07:59	**1.59 (1.30–1.93)**	**1.49 (1.21–1.82)**

*Bivariate logistic analyses between the independent variable and EMS re-contact

**Adjusted for age, sex, urgency level, applicant, time of day, medical specialism, consultation/handover to a GP

^1^Healthcare institution, Obstetrician, Psychiatrist

^2^Fire department, Police

With respect to care process characteristics at the initial contact, cases belonging to the medical specialisms’Pulmonology ‘and ‘Neurology’ were significant more likely to have a re-contact, with the highest likelihood for ‘Pulmonology ‘(OR 2.45 CI 1.67–3.64) followed by ‘Neurology’ (OR 1.81 CI 1.28–2.59). Cases belonging to the medical specialism ‘Traumatology/surgery’ were significant less likely to have a re-contact (OR 0.51 CI 0.34–0.76). Consultation or handover to a GP at the initial contact increased the likelihood of a re-contact (OR 1.25 CI 1.06–1.47).

Regarding run characteristics, initial contacts that occurred during night time compared to those during the day (OR 1.49 CI 1.21–1.82) were more likely to have a re-contact. The urgency level of the initial ambulance run was not associated with a re-contact. Finally, an unknown applicant seems protective for a re-contact (OR 0.78 CI 0.64- 0.95).

### Characteristics of EMS re-contacts

For 984 re-contacts the outcome of the ambulance re-contact was analysed. A total of 661 re-contacts, which remained after omitting initial cases with incomplete data, were further analysed on patient-, care process- and run characteristics (Table 3). The majority of patients after the re-contact was conveyed to the emergency department (ED) or Cardiac Care Unit (CCU) (68%, 665/984), and a quarter ended in non-conveyance again (26%, 258/984). Only 0.5% (5/984) of the patients was found dead or died during treatment at this second contact. Most re-contacts were related to the medical specialism ‘Internal Medicine’ (208/661, 31%). In 51% (340/661) of the cases the medical specialism corresponded between the first and second contact, 31% (204/661) did not correspond. Due to missing data of the re-contact, this was unknown for 18% of the cases. The GP was applicant in most re-contacts (222/661, 33%) and a re-contact occurred in half of the cases during daytime (333/661, 50.5%). The urgency level with which the re-contact is dispatched divided among less urgent levels of urgency A1; 50% (331/661), A2; 35% (229/661) and B; 15% (101/661).

**Table 3:** Characteristics of the EMS re-contact

Variable	EMS re-contact Second contact
Outcome of the re-contact	N = 984
ED/CCU	665 (68%)
Non-Conveyance	258 (26%)
Direct hospital admission	42 (4%)
Outpatient clinic	14 (1.5%)
Death	5 (0.5%)
Care-process characteristics	
Medical specialism	N = 661
Cardiology	76 (14%)
Surgery/traumatology	30 (5.5%)
Pulmonology	68 (12.5%)
Neurology	86 (16%)
Internal medicine	208 (38%)
Pediatrics (including neonatology)	19 (3.5%)
Psychiatry (including panic disorders)	52 (9.5%)
Gynecology/obstetrics	5 (0.5%)
Missing	117 (18%)
Medical specialism same as initial contact	
Yes	340 (51%)
No	204 (31%)
Unknown	117 (18%)
Run Characteristics	
Urgency level	
A1 (arrival < 15 min	331 (50%)
A2 (arrival < 30 min)	229 (35%)
B (planned ambulance care)	101 (15%)
Applicant	
Citizen/112	199 (30%)
General practitioner	222 (33.5%)
GP out-of-hours service	66 (10%)
Other healthcare professional^1^	43 (6.5%)
Non-healthcare professional^2^	12 (2%)
Unknown	118 (18%)
Time of day	
Day 08:00–17:00;	333 (50.5%)
Evening, 17:01–23:59;	207 (31%)
Night, 00:00–07:59	121 (18.5%)

^1^Healthcare institution, Obstetrician, Psychiatrist

^2^Fire department, Police

## Discussion

The aim of this study was to investigate the incidence of re-contacts within 72 h following non-conveyance, to analyse the characteristics of non-conveyance cases with a re-contact with respect to patient-, care process- and run characteristics, and to identify which characteristics are independently associated with re-contacts.

Non-conveyance includes potential risks in which patient safety may be compromised. Re-contacts could therefore be an indicator of patient safety and quality of ambulance care. In our study the incidence of EMS re-contacts within 72 h is low (5%), and comparable to other studies [5, 11, 23]. Additionally, our data shows, that in case of a re-contact, such contacts are less urgent compared to the initial contact and more than a quarter ends in a second non-conveyance decision. Although a cut-off point for re-contacts to determine non-conveyance safety is lacking, our results suggest that non-conveyance is relatively safe and an appropriate decision. However, the validity of re-contacts as quality indicator for non-conveyance is questionable. Although re-contacts can suggest shortcomings and misjudgements in care, re-contacts may also result from reasons beyond healthcare deficiencies, such as deteriorating illness, recurrence of the medical event or a completely unrelated illness. In addition, re-contacts may also reflect planned EMS care [29]. Therefore, a patient's need for healthcare after a non-conveyance decision is not a straightforward indicator of an inappropriate non-conveyance decision [30]. Our study supports such conclusion.

The study identified independent patient-, care process- and run characteristics associated with re-contacts. With respect to patient characteristics, age > 65 years and male gender refers to a higher likelihood to have a re-contact. Our results are consistent with other studies within ambulance and ED-settings, emphasizing the importance of considering age and gender in non-conveyance decisions [20, 21, 23, 31–35]. The association between age and re-contact may be attributed to fragility, complex symptoms, comorbidities, medications, and the high impact of physiological changes in elderly patients. The explanation for the association between male gender and re-contact is unknown, needing further research.

In the context of the care process, regarding medical complaints related to re-contact, pulmonary and neurological complaints have a higher likelihood of a re-contact, whereas complaints related to trauma have a lower likelihood. Similar findings were reported earlier, where medical events, particularly those related to respiratory issues, were more likely to have a re-contact than accident-related events [23]. This might be due to the fact that medical events like pulmonary and neurological issues are more susceptible to recurrence, exacerbations or deterioration over time, because these conditions tend to be chronic or progressive.

With respect to other care process characteristics, non-conveyance cases that are handed over to the GP or in which the GP is consulted appeared on our study to be more likely to have a re-contact. Similar findings were reported in a recent study where physician phone consultation was associated with a higher rate of ED and hospital admissions as well as increased mortality [36]. GP involvement could be for various reasons; there may be an unclear ambulance clinical assessment outcome requiring additional medical expertise, required additional physical examinations by a medical professional, or the necessity for a longer term follow-up policy [37]. As explanation that a GP consultation in the initial contact is associated with a higher likelihood for a re-contact, we speculate that it may be due to a different assessment of the GP than the ambulance professional, or that an agreed medical policy fails to work or that the patient's health condition deteriorates. Further investigation is needed to understand and explore the factors underlying this association.

As for run characteristics, night-time non-conveyance cases are more likely to result in re-contacts. These results are in line with previous research [11, 36]. This might be due to different factors influencing the conveyance-decision during night time. For instance, previous research showed that 73% of the patients conveyed to the ED during night time, did not receive diagnostics or treatment before daytime [38]. Ambulance professionals might incorporate this lag-time in their decision-making. Also, the non-availability of the patient’s own GP might influence decision-making. Furthermore, it is possible that patients present themselves with different complaints during night time, or are less willing to be conveyed during night time. As insight is lacking, further research should focus on non-conveyance decisions during night time.

In our study it is observed that the medical specialism of the re-contact remains consistent with the medical specialism of the initial contact in most cases. This could suggest a similar medical cause in both contacts. However, medical specialism does not separate different diseases (e.g., asthma and pneumonia). On the other hand, a different medical specialism does not necessarily imply an unrelated nature of complaints between the first and second contact. Therefore, further in-depth research should explore the similarity of medical complaints between both contacts and reasons for re-contacts.

The majority of patients with a re-contact are conveyed to the emergency department (ED) or cardiac care unit (CCU), a pattern that resonates with findings from other studies [23]. This tendency can be attributed to the GP re-evaluation or prompted by instances of symptom recurrence or persistent complaints, leading to a new 112 call. The very act of reaching out to EMS for a second time is deemed sufficient grounds for an evaluation at the ED or CCU. The subsequent course of events following admission to the ED or CCU is relevant, but beyond the scope of our study.

### Limitations and strengths

First, our study is limited by its retrospective design, relying on existing data. A second limitation is that our data is limited to information from EMS only, with no input from GPs or EDs. Therefore, we lack data on the subsequent progression of medical situations, final diagnoses or mortality rates. A third limitation of this study pertains to a notable percentage of missing data regarding the variable medical specialism. Consequently, not all non-conveyance cases could be included in the analysis. While the group with missing data did not significantly differ from complete cases across most variables, bias cannot be excluded. A final limitation is the potentially limited generalizability of the data across regions and countries. Variations in demographics, socio economic status, population density, culture, and healthcare systems might affect the clinical profile of non-conveyance cases with a re-contact. Finally, it should be noted that our study is based on data from 2022, during which the initial months included the aftermath of the COVID-19 pandemic, warranting consideration in the interpretation of our results.

A particular strength is that our study is the first Dutch study that studied the characteristics of non-conveyance cases with a re-contact within 72 h and it represents an important initial step towards better comprehension of these cases. It also includes information about large numbers of cases, with data from a hole year and of two different regions. We further were able to come up with a clinical profile of non-conveyance cases at risk for a re-contact. By identifying independently associated factors this study provides valuable insights depicting system performance. Results may be a start to inform non-conveyance decision-making, adjust EMS curricula, and develop guidelines and protocols.

### Further research and recommendations

Our study emphasizes the need for future (prospective) research and more comprehensive data collection and data points. Such research could profit from classifying the reasons of a re-contact according to a classification used in studies about the emergency department [39–41]. Classifying into illness- related (e.g., disease progression), patient-related (e.g., non-compliance with medical advice) and healthcare professional (e.g., misjudgements) or system-related reasons of a re-consult, provides valuable information about the content of a re-contact and the relationship between the initial and the subsequent contact. Moreover, this enables a more robust assessment of healthcare quality, which we believe is crucial when utilizing re-contacts as a quality indicator. Scientific knowledge could also be improved with research information on decision making of GPs and at the ED, as well as information on medical complaints, diagnosis, disease progression and hospital admission information. This information is valuable in the evaluation of the provided ambulance care. In terms of improving the clinical profile, the incorporation of more factors, including specific working diagnose and vital signs [42], as well as medical history and socioeconomic status, holds promise for enhancing the optimization of the clinical profile for non-conveyance cases at risk for EMS re-contact. Finally, there is accumulating evidence of the determinants of non-conveyance and re-contact. This opens the opportunity for meta-analysis and system comparison.

## Conclusions

The study identified a low overall incidence of EMS re-contacts, indicating that non-conveyance within the Dutch EMS system is relatively safe. Factors related to the patient, the medical condition, the healthcare system, and time points were identified. Moreover, the data challenge the qualification of re contact as merely a quality issue. Since re-contacts are higher for age above 65, male gender, and medical complaints within the specialisms of 'Pulmonology' and 'Neurology', after consultation or handover to a GP, and in cases occurring at night it may be possible in the future to profile patients at higher risk for an EMS re-contact after non-conveyance.

These findings inform non-conveyance decision-making, and could serve as a starting point to adapt EMS curricula, and develop guidelines and protocols, in order to improve the quality of healthcare within the EMS system.

## Data Availability

The datasets used and/or analysed during the current study are available from the corresponding author on reasonable request.

## Abbreviations

EMS: Emergency Medical Services
GP: General Practitioner
ED: Emergency Department
IQR: Interquartile range
CI: Confidence interval
CCU: Cardiac care unit
